# Protein profiling reveals inter-individual protein homogeneity of arachnoid cyst fluid and high qualitative similarity to cerebrospinal fluid

**DOI:** 10.1186/2045-8118-8-19

**Published:** 2011-05-20

**Authors:** Magnus Berle, Ann C Kroksveen, Øystein A Haaland, Thin T Aye, Jill A Opsahl, Eystein Oveland, Knut Wester, Rune J Ulvik, Christian A Helland, Frode S Berven

**Affiliations:** 1 Institute of Medicine, University of Bergen, 5021 Bergen, Norway; 2 Proteomics Unit at University of Bergen (PROBE), Department of Biomedicine, University of Bergen, 5021 Bergen, Norway; 3 Department of Mathematics, University of Bergen, 5008 Bergen, Norway; 4 Department of Biomedicine, University of Bergen, 5021 Bergen, Norway; 5 Department of Surgical Sciences, University of Bergen, 5021 Bergen, Norway; 6 Department of Neurosurgery, Haukeland University Hospital, 5021 Bergen, Norway; 7 Laboratory of Clinical Biochemistry, Haukeland University Hospital, 5021 Bergen, Norway; 8 The Norwegian Multiple Sclerosis Competence Centre, Haukeland University Hospital, Bergen, Norway

## Abstract

**Background:**

The mechanisms behind formation and filling of intracranial arachnoid cysts (AC) are poorly understood. The aim of this study was to evaluate AC fluid by proteomics to gain further knowledge about ACs. Two goals were set: 1) Comparison of AC fluid from individual patients to determine whether or not temporal AC is a homogenous condition; and 2) Evaluate the protein content of a pool of AC fluid from several patients and qualitatively compare this with published protein lists of cerebrospinal fluid (CSF) and plasma.

**Methods:**

AC fluid from 15 patients with temporal AC was included in this study. In the *AC protein comparison experiment*, AC fluid from 14 patients was digested, analyzed by LC-MS/MS using a semi-quantitative label-free approach and the data were compared by principal component analysis (PCA) to gain knowledge of protein homogeneity of AC. In the *AC proteome evaluation experiment*, AC fluid from 11 patients was pooled, digested, and fractionated by SCX chromatography prior to analysis by LC-MS/MS. Proteins identified were compared to published databases of proteins identified from CSF and plasma. AC fluid proteins not found in these two databases were experimentally searched for in lumbar CSF taken from neurologically-normal patients, by a targeted protein identification approach called MIDAS (Multiple Reaction Monitoring (*MRM*) initiated detection and sequence analysis).

**Results:**

We did not identify systematic trends or grouping of data in the *AC protein comparison experiment*, implying low variability between individual proteomic profiles of AC.

In the *AC proteome evaluation experiment*, we identified 199 proteins. When compared to previously published lists of proteins identified from CSF and plasma, 15 of the AC proteins had not been reported in either of these datasets. By a targeted protein identification approach, we identified 11 of these 15 proteins in pooled CSF from neurologically-normal patients, demonstrating that the majority of abundant proteins in AC fluid also can be found in CSF. Compared to plasma, as many as 104 proteins in AC were not found in the list of 3017 plasma proteins.

**Conclusions:**

Based on the protein content of AC fluid, our data indicate that temporal AC is a homogenous condition, pointing towards a similar AC filling mechanism for the 14 patients examined. Most of the proteins identified in AC fluid have been identified in CSF, indicating high similarity in the qualitative protein content of AC to CSF, whereas this was not the case between AC and plasma. This indicates that AC is filled with a liquid similar to CSF. As far as we know, this is the first proteomics study that explores the AC fluid proteome.

## Background

Arachnoid cysts (AC) are benign intracranial lesions with a reported prevalence in the adult population of up to 1% [1,2]. AC can be found all along the cranio-spinal axis, but have a marked predilection for the temporal fossa [3]. Anatomically, ACs are formed by a splitting of the arachnoid mater (AM) creating a potential space that when filled with fluid appears as a cyst [4,5]. Temporal ACs are classified according to Galassi *et al*. [6]. Briefly, a type I cyst is small, biconvex, and located at the anterior temporal pole. A type II cyst involves the proximal and intermediate segments of the Sylvian fissure, and a type III cyst involves the entire Sylvian fissure and has often a marked radiological mass effect. The cyst wall is composed of non-neoplastic arachnoid cells with a capacity to secrete fluid [7,8] that slightly differs in chemical composition from that of cerebrospinal fluid (CSF) [9]. The genetic profile of temporal AC membranes [10] indicates that these cysts represent a homogenous condition, but the underlying cause of AC formation is unknown. Further analyses of the cyst fluid with proteomics, the large-scale studies of proteins, might give indications of the aetiology of AC and thus shed further light on the mechanisms underlying fluid secretion and transport.

One aim of this study was to compare the protein content of cyst fluid from temporal AC of different individuals using proteomics (*AC protein comparison experiment*). Our hypothesis was that AC is a homogenous condition, and that we could identify a similar protein profile from AC from different patients. Homogenous protein content between AC fluid from different patients would point towards similar filling mechanisms for the examined patients. Large differences in some patients would indicate different filling mechanisms in these patients. Such a difference, if present, would be revealed by a label-free proteomics comparison approach. Mass spectrometry-based label-free approaches are commonly used for semi-quantitative comparison of complex protein samples [11].

Another aim of the study was to identify the major proteins present in AC fluid and examine if these proteins also appeared in CSF and plasma (*AC proteome evaluation experiment*). For the proteins that did not appear in the CSF and plasma protein databases, we used a targeted mass spectrometry protein identification approach referred to as MIDAS in an attempt to identify these AC fluid proteins in CSF. The protein content of AC fluid is largely unknown, but it has been shown to have reduced protein content relative to that of CSF from the same patient, as evaluated by clinical chemistry [9]. In a similar study, Sandberg *et al. *[12] studied the clinical chemistry of AC fluid in pediatric patients. We did not have specific hypotheses on the degree of similarity of AC fluid to CSF or plasma, as this has not been thoroughly demonstrated in literature. This AC fluid protein identification part of our study and comparison with CSF and plasma might give information about the origin of the AC fluid and the mechanisms of filling and sustaining of AC.

## Methods

### Participants and collection of AC fluid and CSF

15 patients (six male, nine female, age 22-77 y) with unilateral, temporal AC were included in the study. AC fluid was collected during surgery for AC at Haukeland University Hospital (Bergen, Norway). The patients' characteristics are summarized in Table 1. One patient (No. 3) had previously been operated for a chronic subdural hematoma, most probably caused by the cyst [13], and another patient (No. 4) had undergone previous surgery for the AC. The other patients had no previous history of intracranial hematomas or surgery. The sample collection and handling protocol used in this study have been described in detail elsewhere [9,14]. Briefly, AC fluid was collected during elective surgery for AC (craniotomy with fenestration and extirpation of the cyst) by puncturing the dura with a 23G, 25 mm long syringe needle using an Optidynamic^® ^spinal fluid manometer (Mediplast AB, Malmo, Sweden) by siphoning through a burr hole before the craniotomy/opening of the dura. This procedure ensures that CSF does not contaminate the collected AC fluid: The remaining fluid in the cyst was removed by suction during opening of the cyst wall. The collected AC fluid was centrifuged at 450 × *g *for 5 min to remove cells and cell debris, and the supernatant was aliquoted and frozen at -80°C. Deviations and observations on individual sample material were noted on sampling. During this sampling, slight hemolysis was observed in one patient sample (No 12). The CSF used for the *targeted AC protein identification experiment *was collected under informed consent as lumbar CSF before spinal anaesthesia in patients undergoing lower extremity orthopedic surgery from neurologically healthy individuals. The CSF was handled by the same protocol as for the AC fluid, and the CSF used in this experiment was pooled from 11 individuals.

**Table 1:** Table of arachnoid cyst patients in study, with age/sex, sidedness, Galassi-stage [6] and remarks.

Patient	Age (yrs), sex	Side	**Galassi-stage **[6]	Remarks
1	26, f	Left	2	
2	43, m	Left	2	
3	58, f	Left	3	Old haematoma
4	34, f	Left	2	Reoperation
5	22, f	Right	1	
6	36, f	Right	2	
7	35, f	Right	2	
8	77, f	Left	1	
9	42, f	Left	1	
10	60, m	Left	2	
11	56, m	Right	2	
12	25, m	Left	1	Slight observed haemolysis
13	30, f	Left	1	
14	37, m	Left	2	
15	63, m	Left	2	

### Ethics

Patients were recruited by the responsible surgeon and signed a written informed consent. This project was approved by the Regional Committee for Medical and Health Research Ethics (REK) of Western Norway (approvals REK 70.03, NSD 9634, REK 151.06 and REK 2009/1885).

### Chemicals

Trypsin was purchased from Promega (Fitchburg WI, USA). Urea, acetonitrile (ACN), formic acid (FA), calcium chloride (CaCl_2_), iodoacetamide (IAA) and dithiothreitol (DTT), potassium phosphate monobasic (KH_2_PO_4_), potassium chloride (KCl), water and trifluoroacetic acid (TFA) were purchased from Sigma-Aldrich (St. Louis MO, USA). Water and ACN were of HPLC quality.

### Sample preparation and protein digestion

The protein concentration in AC fluid was measured using a Qubit™ fluorometer (Life Technologies, Carlsbad CA, USA). AC fluid was concentrated and desalted using Amicon 3 kDa molecular weight cut-off filters (Millipore, Billerica, MA, USA) and dried in a vacuum concentrator (Eppendorf, Hamburg, Germany). The proteins were digested into peptides using in-solution digestion, as follows: The dried protein pellet was dissolved in 6 M Urea and 100 mM DTT and incubated for 1 h at 37°C. Cysteins were alkylated using 200 mM iodoacetamide and the samples were incubated for 1 h at 37°C. Chymotrypsin activity was inhibited by adding 2 mM CaCl_2 _the proteins were digested to peptides over night using a protein:trypsin ratio of 1:50. Each sample was acidified using 5% TFA to quench the digestion activity, followed by drying the sample completely in a vacuum concentrator.

### Strong cation exchange (SCX) chromatography

The samples were dissolved in 120 μL of SCX loading buffer (5 mM KH_2_PO_4_, 25% ACN, 0.05% FA, pH = 3) and fractionated by SCX chromatography using an Ultimate 3000 LC system (Dionex, Ultimate, Sunnyvale, CA, USA) equipped with a BioBasic SCX column (150 mm × 2.1 mm, 5 μm, Thermo Scientific, Ontario, Canada). The peptides were eluted in SCX elution buffer (500 mM KCl, 5 mM KH_2_PO_4_, 25% ACN, 0.05% FA, pH 3.0) over 55 min with a flow rate of 0.2 mL/min. A total of 28 SCX fractions were collected. The first two fractions (SCX fraction 1 and 2) were collected with 5.5 min intervals (first 11 min) and the last fraction (fraction number 28) was collected over the last 5 min. Fractions number 3-27 was collected with 1.5 min intervals. After collection, each SCX fraction was vacuum concentrated to dryness.

### Sample clean-up

The samples were desalted using a 96 well reverse phase Oasis^® ^HLB μElution Plate 30 μm (Waters, Wilford, MA, USA). The wells in the μElution plate were conditioned with solvent B (80% ACN, 0.1% FA) and thereafter washed twice with solvent A (0.1% FA). The peptides were re-suspended in solvent A, added to the μElution plate, and washed thrice with solvent A before the peptides were eluted twice using solvent B. One-minute centrifugation at 200 × *g *was used for all centrifugation steps except for addition of sample where 3 min at 150 × *g *was used. The samples were concentrated to dryness under vacuum and frozen at -80°C prior to mass spectrometry (MS) analysis.

### Mass spectrometry

In the *AC protein comparison experiment*, the peptides were dissolved in 0.1% FA, and 4 μL (1.6 μg) of the sample was injected onto a 40nL enrichment column (Zorbax 300SB C18 5 μm, Agilent Technologies, Santa Clara, CA, USA) at a flow rate of 3 μL/min using 3% ACN, 0.1% TFA. The separation column (0.0075 × 43 mm Zorbax 300SB C18 5 μm, Agilent Technologies) was used with the following gradient and a flow rate of 300 nL/min using solvent A (0.1% FA) and solvent B (90% ACN, 0.1% FA): 3-15% solvent B for 3 min, 15-45% solvent B for 42 min, 45-90% solvent B for 5 min and back to 3% solvent B after 5 min. Both columns were integrated in a CHIP (Agilent Technologies, Santa Clara, CA, USA), and an 1100 cap/nano HPLC coupled to a chip-cube-LC/MSD XCT Plus ion trap mass spectrometer (Agilent Technologies, Santa Clara, CA, USA) was used for separation and analysis, respectively. MS data was acquired using the AutoMS2 mode of the three precursors with highest intensity active exclusion for 1 min.

For the *AC proteome evaluation experiment*, the peptides were dissolved in 0.1% FA and 5 μL of the sample was injected to the analytical fused-silica capillary column (15 cm long, 75 μm i.d.) packed with Reprosil-Pur 3 μm C18 resin (Dr. Maisch, Ammerbuch-Entringen, Germany). The settings for LC were: Trap column: 2% ACN, 0.1% FA with a flow rate of 25 μL/min. Analytical column: solvent A was 0.1% FA and solvent B was 90% ACN, 0.1% FA. The flow rate was 0.288 μL/min with the following gradient: 5-12% solvent B for 2 min, 12-30% solvent B for 48 min, 30-50% solvent B for 20 min, 50-95% solvent B for 1 min and 95% solvent B was kept constant for 5 min before regeneration of the column for 24 min. The nano-HPLC system (Dionex, Ultimate, Sunnyvale, CA, USA) was coupled to an Ultima Global ESI-Q-TOF mass spectrometer (Waters, Wilford, MA, USA). The scan area for the MS survey scan was *m/z *300-1500 with automatic fragmentation of the three ions with highest intensity. All the data was acquired in data dependent mode.

For the *targeted AC protein identification experiment*, the tryptic peptides of CSF from neurologically normal patients were dissolved in 0.1% FA and 1 μL was injected into the Q-TRAP 5500 (AB Sciex, Foster City, CA, USA) coupled to a nano-HPLC system (Dionex, Ultimate, Sunnyvale, CA, USA). The targeted mass spectrometry analysis was done using the MIDAS (Multiple Reaction Monitoring (*MRM*) initiated detection and sequence analysis) workflow [15] selecting a minimum of 3 peptides per protein, and three transitions per peptide based on information from *in silico *digestion. The instrument settings were 15 ms dwell time with approximately 3.5 s cycle time for 100 transitions per method in four analyses. MRM was then used as a survey scan in information dependent acquisition (IDA) to detect specific peptide peaks, and each resulting MRM peak was examined by two full MS/MS-scans to obtain sequence verification of the hypothesized peptide.

### Mass spectrometry data analysis

For the *AC protein comparison experiment*, the raw data was processed with the Spectrum Mill search engine (Rev A.03.03.084) (Agilent Technologies, Santa Clara, CA, USA) using Carbamidomethylation (C) as fixed modification. The precursor mass tolerance was set to 2.5 Da with a product mass tolerance of 0.7 Da, and two trypsin miss-cleavages were allowed. The default autovalidation settings were applied for both protein and peptide level validation. Briefly, peptides were accepted at charge +2 if score >11 and%SPI >60 and at charge +3 if score >13 and%SPI >70. Proteins with a score >20 were accepted. A threshold of 2 was set both for peptides and proteins for the forward-reverse score and the rank 1-2 score, except for charge +2 with score >6 and%SPI > 90 where the threshold was 1.

For the *AC proteome evaluation experiment*, the mass list was extracted into PKL files with Masslynx (Waters, Wilford, MA, USA) and the PKL files were merged into a single MGF file using PklFileMerger [16]. All MS/MS data were searched using the MASCOT (version 2.2.2) software platform (Matrix Science, London, UK) against the IPI-human database (v3.69, 174784 entries). Missed cleavages were set to one using trypsin as the enzyme. Carbamidomethyl (C) was set as fixed modification whereas Oxidation (M) was set as variable modifications. The peptide tolerance was set to 40 ppm and the MS/MS tolerance was set to 0.6 Da. Protein organization and redundancy reduction was done using the Scaffold software (v3.00.02 [17]). Peptide and protein identifications were accepted when the probability of correct identification was greater than 95%, with a minimum of two identified peptides per protein.

To verify peptide specificity in designated protein targets in the *targeted AC protein identification experiment*, MS/MS spectra were extracted using Analyst software (version 1.5) (Life Technologies, Carlsbad CA, USA). The selected spectra were searched using the MASCOT (version 2.3.0) software platform (Matrix Science) against the IPI-human database (version 3.78, 302626 entries) using precursor mass and MS/MS tolerance at 0.2 Da. Carbamidomethyl (C) was set as fixed modification and 0 miss cleavages were chosen.

The protein list from the AC fluid pool created in the *AC proteome evaluation experiment *was compared to published proteome libraries of CSF [18] and plasma [19]. Database comparison was performed using ProteinCenter, v3.2.0.9 (Proxeon, Odense, Denmark). The grouping of proteins and analysis of molecular function and biological processes was performed in ProteinCenter, where gene ontology terms linked to specific proteins are obtained using mappings from the Gene Ontology Consortium website [20].

### Statistical methods

Principal component analysis (PCA) is a mathematical method that transforms a number of possibly correlated variables into a smaller number of uncorrelated variables called principal components, with the objective of improved understanding of the data [21]. The algorithm of PCA gives the weight of each principal component, meaning the percentage of variation in the dataset explained. In the *AC protein comparison experiment*, the protein content between individual samples was compared based on spectral intensities. The spectral intensities in each sample were normalized based on the total sum of all intensities in that sample relative to a chosen reference samples. The protein hits from the *AC protein comparison experiment *were sorted according to protein score. To check for possible outliers, we first did a PCA including all identified proteins in the analysis, and plotted the patients according to the two first principal components. Next, we compared the samples based on the 50 proteins with highest protein score to avoid large influence from proteins identified in a low number of samples. Finally, we used a sub-sampling scheme [22,23] consisting of two steps:

1 - select 50 proteins at random and measure the distance normalized to standard deviation from the origin of each patient and 2 - repeat the first step 1000 times, and calculate the average distance from the origin over the 1000 replications.

This analysis was designed to reveal the degree to which any particular sample is divergent from the rest. Statistics was performed in the statistical software package R, version 2.11.1. (The R Foundation for Statistical Computing, Vienna, Austria).

## Results

**Figure 1:**
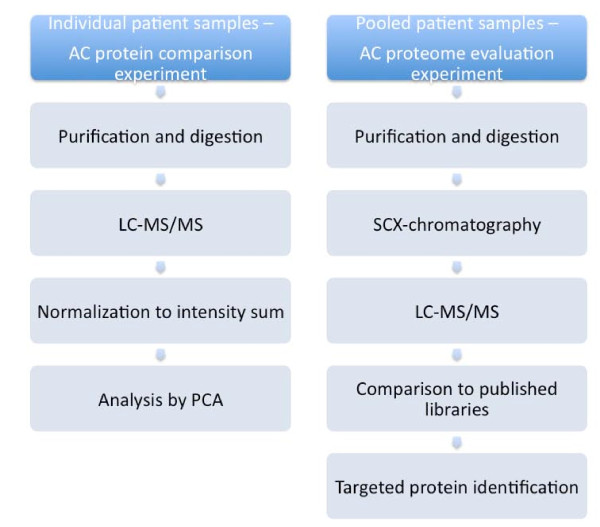
**Flow charts of the experimental procedure for the arachnoid cyst protein comparison experiment and for the arachnoid cyst proteome evaluation experiment**.

A flow chart of the two main experiments conducted in this study, the *AC protein comparison experiment *and the *AC proteome evaluation experiment *is given in Figure 1.

### The AC protein comparison experiment

**Figure 2:**
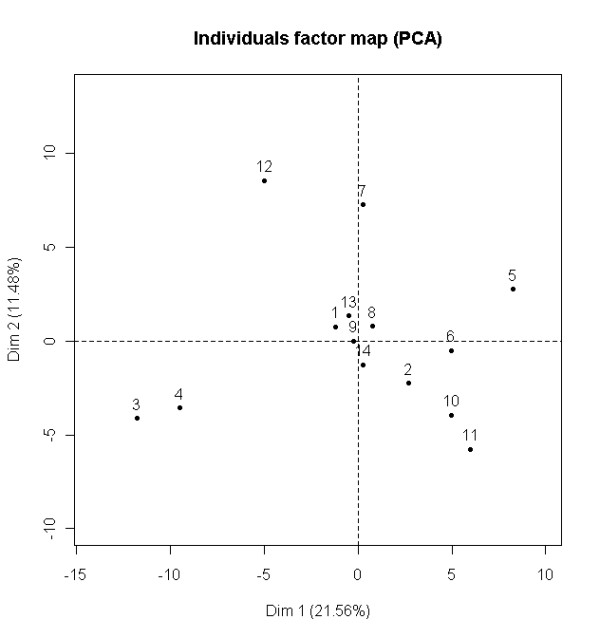
**Arachnoid cyst protein comparison experiment (all proteins)**. Principal component analysis (PCA) of individual AC fluid LC-MS/MS results, normalized to sample sum. The figure is based on evaluation of all 139 proteins. Axis labels are weights of axes and the amount of total variance explained by the PC. The percentage of variance explained by the first two PCs was 33%. Observe that patients 3 and 4, as well as patient 12 were apart from the other patients.

**Figure 3:**
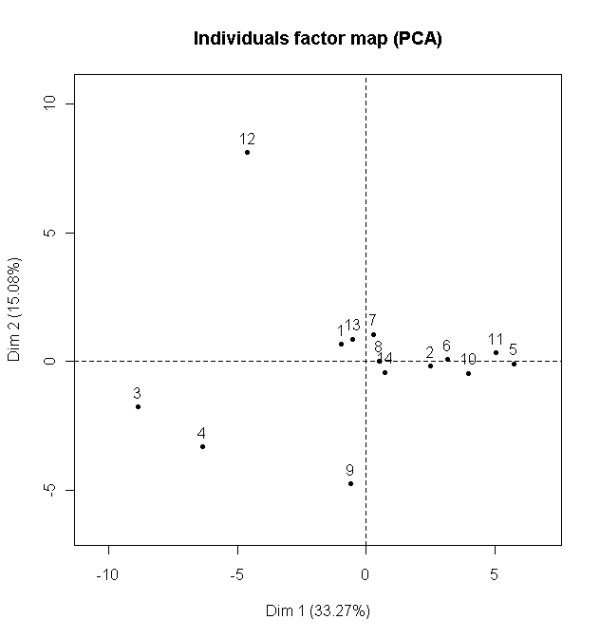
**Arachnoid cyst protein comparison experiment (top 50 proteins)**. Principal component analysis (PCA) of individual AC fluid LC-MS/MS results, normalized to sample sum. The figure is based on evaluation of top 50 proteins on protein score. Axis labels are weights of axes, the amount of total variance explained by the PC. The percentage of variance explained by the first two PCs is 48%. Observe that patients 3 and 4, as well as 12 and possibly 9 were apart from the other patients.

In the *AC protein comparison experiment *the protein contents of AC fluid from 14 patients (patients 1-14) were semi-quantitatively compared using a label-free proteomics approach to determine the protein homogeneity across the different patients. The peptides from each of the 14 patients were analyzed on an ion-trap mass spectrometer, and the protein extracted ion chromatographic (XIC) intensity values, extracted from the Spectrum Mill searched data, were compared between the different patients using PCA. In total, 139 proteins were identified from the 14 different patients. When all 139 proteins were compared using PCA, the two first principal components (PC) explained 33% of the total variation in the dataset (Figure 2). As low scoring proteins with few associated spectra can lead to more inaccurate semi-quantitative measurements, we also compared the 50 proteins with highest protein scores. For this dataset, the two first PCs explained 48% of the total variation in the dataset (Figure 3). This was a larger degree of explained variation than the model based on all 139 proteins. A comparison based on the 50 highest scoring proteins gave a clearer separation in absolute distance from the origin for samples from patients 3 (old hematoma), 4 (previous operation) and 12 (slight observed hemolysis), as well as possibly patient 9, than for the other patients. To check whether or not this separation was due to a single or a few proteins, we performed a sub-sampling scheme. Selecting 50 of the 139 proteins at random for 1000 iterations and evaluating absolute distance in standard deviations from the origin showed that patients 3, 4 and 12, the samples presumed to be different, are consistently further away from the sample mean than the rest of the patients (Table 2), while this was not the case for patient 9. Disregarding patients 3, 4 and 12, the dataset of individual patient's AC fluid samples presents little systematic variation. We did not find that a few specific proteins create the separation of patients 3, 4 and 12 from the others. There was no apparent sub-grouping or trends in the data. The lack of systematic trends is an indication of homogenous sample material, thus suggesting that AC is a homogenous condition as evaluated by proteomics.

**Table 2:** Arachnoid cyst fluid protein comparison experiment: distance in dataset standard deviation from the origin for each patient.

Patient	All protein scores - figure 2	50 highest protein scores - figure 3	1000 iterations of 50 randomly-selected protein scores
1	0.3	0.3	0.5
2	0.7	0.6	0.7
3	2.3	2.2	2.1
4	1.9	1.9	1.8
5	1.6	1.4	1.7
6	0.9	0.7	1.0
7	1.8	0.4	1.3
8	0.2	0.1	0.8
9	0.0	1.7	0.7
10	1.3	1.0	1.2
11	1.8	1.2	1.5
12	2.3	3.0	1.8
13	0.3	0.3	0.5
14	0.3	0.2	0.4

Evaluation of all 139 proteins (Column 2), the top 50 proteins on protein score (Column 3) and an average over 1000 reiterations of PCA performed on pulling 50 of the 139 proteins in a random sub-sampling scheme (Column 4). The table show that the standard deviations of patient 3, 4 and 12 were higher than for the others. This was not the case for patient 9. The values represent the distance from each individual patient to the mean of all patients, using the first two principal components from the PCA. The results were normalized with respect to sample standard deviations.

### The AC proteome evaluation experiment

**Figure 4:**
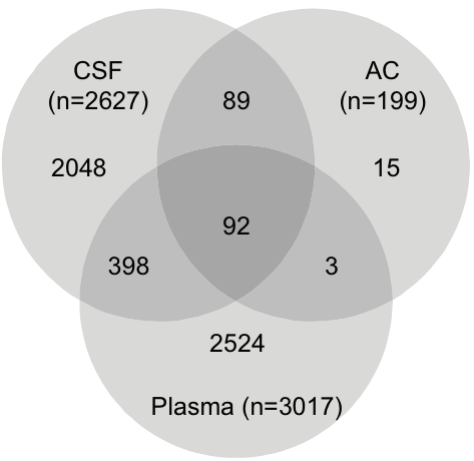
**Venn-diagram showing the protein IPI-identity overlaps between arachnoid cyst fluid, previously recorded CSF **[18] and plasma [19]. Each large circle defines the content of the three individual dataset. The numbers indicate proteins in each group, the overlap between larger circles indicate the number of protein identities in common.

**Figure 5:**
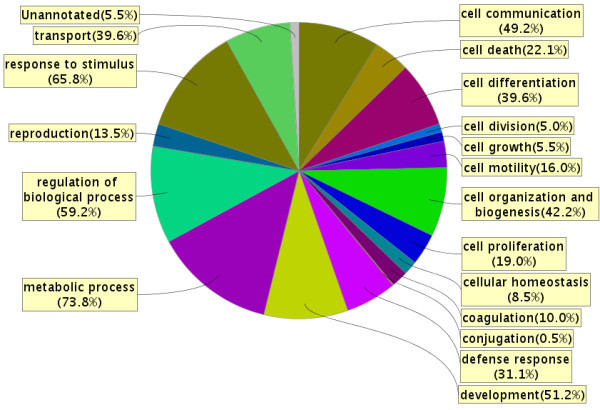
**Biological process involvement of the 199 arachnoid cyst fluid proteins identified, denoted in percentage of proteins**. Note that any one protein may be involved in several processes. Observe that 39.6% of the proteins are involved in transport processes. The figure was made using ProteinCenter, v3.2.0.9.

**Figure 6:**
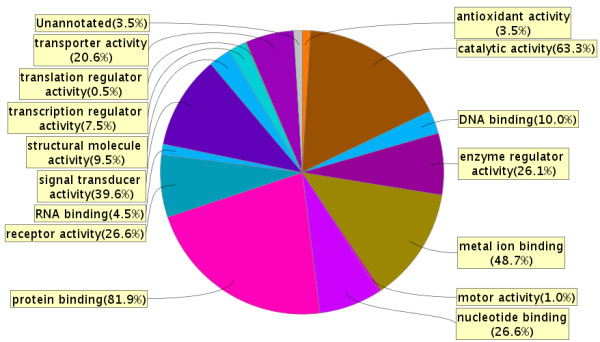
**Molecular functions of 199 arachnoid cyst fluid proteins identified expressed as percentage of proteins**. Note that any one protein may have several functions. Observe that 20.6% of the proteins are involved in transporter activity. The figure was made using ProteinCenter, v3.2.0.9.

**Figure 7:**
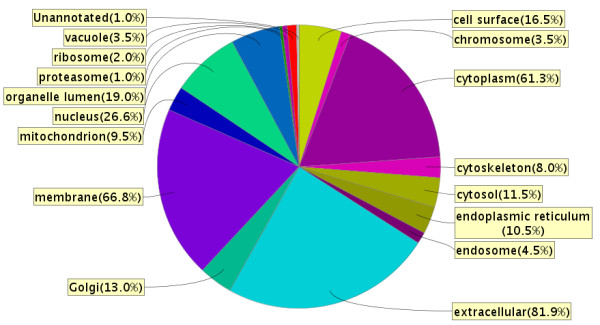
**Cellular component localization of 199 arachnoid cyst fluid proteins identified, expressed as percentage of proteins**. Note that each protein may be identified in several localizations. 81.9% of the proteins are annotated as extracellular proteins, while 66.8% are annotated as membranous proteins. The figure was made using ProteinCenter, v3.2.0.9.

**Figure 8:**
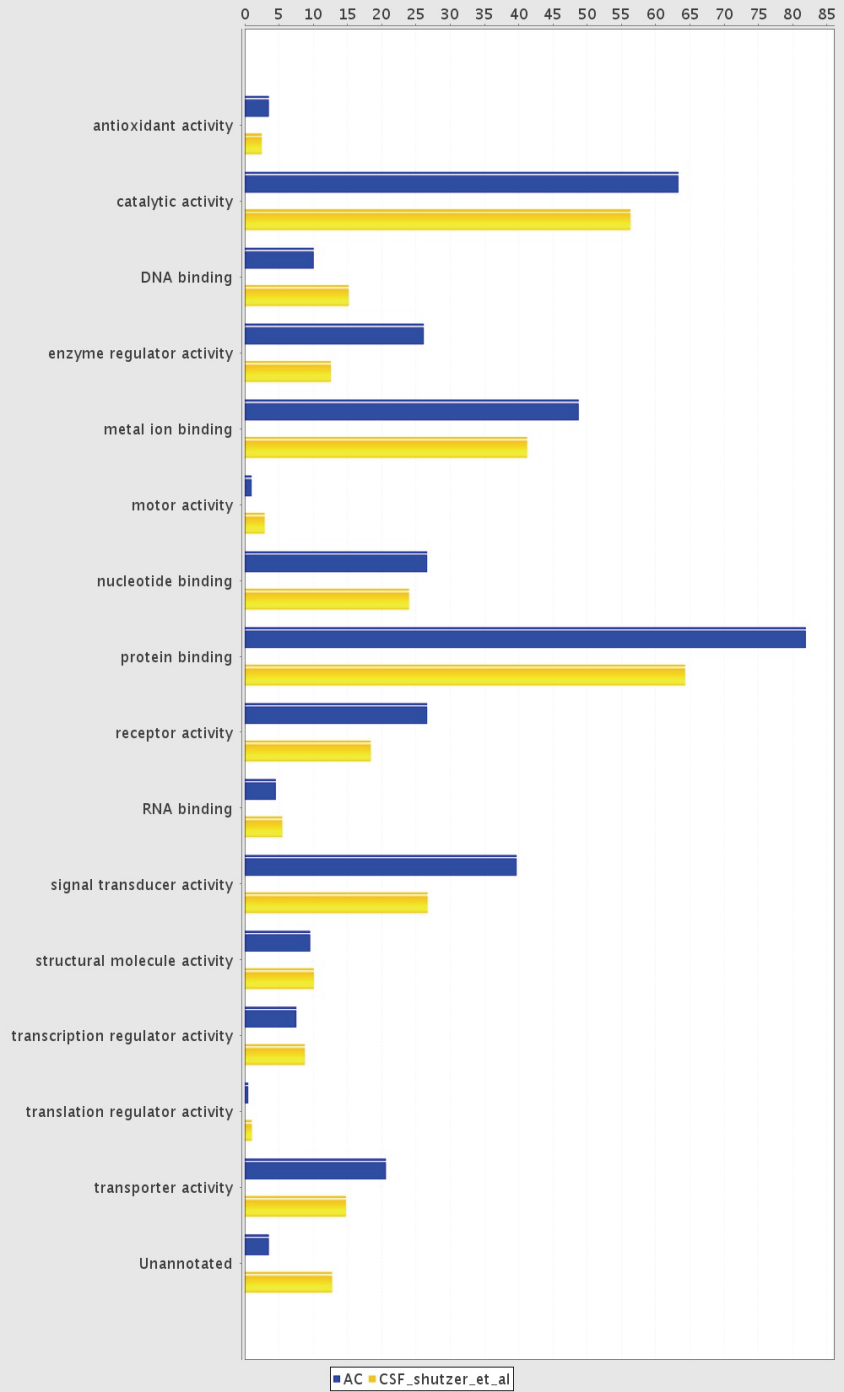
**Comparison of proteins in arachnoid cyst fluid and CSF **[18]**based on molecular function, expressed as percentage of proteins**. Note a large difference in number of proteins detected and used as a basis for creating the figure: 199 proteins in AC and 2627 in the CSF database. The figure was made using ProteinCenter, v3.2.0.9.

In the *AC proteome evaluation experiment*, the aim was to identify the major protein components of AC fluid, and to qualitatively compare these proteins to the proteins previously identified in CSF and plasma and thereby learn more about the composition and origin of AC fluid. Based on the results in the *AC protein comparison experiment*, AC fluid from patients with identified blood protein haemoglobin in the cyst fluid was excluded from this analysis (patients 7, 12, 13 and 14). In this experiment, 10 μg AC fluid from the remaining 11 patients was pooled, digested, fractionated using SCX chromatography, purified and analyzed on a Q-ToF mass spectrometer. From these analyses, 199 proteins were identified in the AC fluid (Additional File 1: List of 199 proteins detected in a pool of AC fluid from 11 patients, with number of peptides and sequence coverage). The identified proteins spanned a large range of MWs, with apolipoprotein C-I being the smallest with a MW of 9.3 kDa, and protocadherin fat 2 being the largest with a molecular weight of 479.3 kDa. The isoelectric point of the proteins ranged from 4.35 to 9.96, represented by cell growth regulator with EF hand domain protein 1 and NANUC-1 heavy chain protein, respectively. The MW and pI calculations were performed using the Compute pI/Mw tool [24]. The 199 identified proteins were compared based on IPI accession numbers to published libraries of CSF [18] and plasma [19] (Figure 4). The database comparison identified 15 proteins that were not reported in CSF or plasma and we identified 11 of these by targeted protein identification in lumbar CSF samples using the MIDAS workflow on a Q-TRAP 5500 mass spectrometer (Table 3). Hence, most proteins identified in AC fluid (195 out of 199) were proven to also be present in CSF or plasma. The four proteins that were not found in the databases were; Isoform 3 of seizure 6-like protein 2, full-length cDNA clone CS0DD006YL02 of neuroblastoma of *Homo sapiens*, isoform 2 of neuroendocrine protein 7B2, and cell adhesion molecule 1 (Table 3). The overlap between AC fluid and plasma only contained 3 supplementary proteins not identified in CSF, whereas CSF and AC fluid had 89 proteins in common but not found in plasma (Figure 4). The 199 identified proteins identified in AC fluid were annotated to a diverse range of biological processes, molecular functions, and sub cellular compartments using the ProteinCenter, v3.2.0.9 software (Proxeon, Odense, Denmark) (Figures 5 and 6 and 7). Of the 199 proteins we identified, 39.6% are involved in transport as a cellular activity (Figure 5), 20.6% are involved in transport activity on molecular function (Figure 6), 81.9% are annotated as extracellular proteins, while 66.8% are membranous proteins (Figure 7). A comparison of molecular functions of the proteins identified in AC and CSF demonstrated a similar distribution (Figure 8). Ten genes have previously been found to be differentially expressed in AC membranes compared to normal arachnoid; NKCC1 [7] and ASGR1, DPEP2, SOX9, SHROOM3, A2BP1, ATP10D, TRIML1, BEND5 and NMU [10]. We did not find the corresponding protein products among the 199 identified proteins in our study.

**Table 3:** Proteins in arachnoid cyst fluid not identified in published libraries of CSF [18] or plasma [19], and peptides identified from targeted identification in samples of normal CSF.

IPI number	Protein name	Molecular weight (KDa)	# peptides identified in CSF
IPI00178854	Contactin-4	113.454	1
IPI00332887	cDNA, FLJ92887, Homo sapiens protein tyrosine phosphatase, non-receptor typesubstrate 1 (PTPNS1), mRNA	54.967	4
IPI00218803	Isoform B of Fibulin-1	77.214	7
IPI00855821	NRXN1-alpha	169.913	4
IPI00018276	Isoform 3 of Seizure 6-like protein 2	97.501	ND
IPI00216250	Cell recognition protein CASPR4	145.660	1
IPI00479708	Full-length cDNA clone CS0DD006YL02 of Neuroblastoma of Homo sapiens (human)	41.273	ND
IPI00022418	Fibronectin	262.625	12
IPI00645363	Putative uncharacterized protein DKFZp686P15220	51.725	7
IPI00470716	Isoform 2 of Neuroendocrine protein 7B2	23.730	ND
IPI00003813	Cell adhesion molecule 1	48.509	ND
IPI00435020	Isoform 2 of Neural cell adhesion molecule 1	94.574	6
IPI00384998	Isoform 7 of Neurofascin	150.027	4
IPI00451624	Cartilage acidic protein 1	71.421	3
IPI00219664	Isoform 2 of Myelin-oligodendrocyte glycoprotein	28.179	1

Proteins that were not found in any of the databases or in the targeted identification experiment is indicated by not detected (ND).

## Discussion

As far as we know, this is the first study of proteomic evaluation of AC fluid. In the *AC protein comparison experiment *we looked for systematic differences or distributions between AC from different patients. The hypothesis underlying this experiment was that the protein profile in AC fluid would be similar between the examined patients, and this was supported by our data. This indicates a common filling mechanism and source for AC fluid in these patients. Previous observations of left side domination in males as well as a predilection for the middle cranial fossa do imply a common origin of AC fluid between patients. One possible method of formation is a defect during the embryological development of the Sylvian fissure [25]. AC could potentially be two or more conditions or subgroups with different filling mechanisms but our proteomics data from the different samples of AC fluid did not support this hypothesis, as no systematic variation in PCA-plots was observed (Figures 2, 3). This finding supports previous experiments performed by clinical chemistry [9] and mRNA [10]. We observed some variation for patients 3, 4 and 12, which can be explained by patient 3 having an old hematoma in close proximity and patient 4 previously operated for the same condition. Patient 12 had a slight observed haemolysis of the AC fluid. We had anticipated a change in the proteome as a consequence of a local trauma and consecutive repair mechanisms.

There was an increase in the percentage variation explained by the first two PCA components when looking at only the 50 highest scoring proteins compared to all 139 proteins. This can be explained by the fact that low scoring proteins often are represented by few peptide observations, which will lead to few values for semi-quantitative measurements, and the uncertainty in the measurements is increased. Hence, focusing only on the proteins with the highest protein scores will give a more accurate picture of the comparison. The method of sub-sampling was useful for evaluation of the stability of a result, such as for the evaluation on whether patient 9 was an actual outlier relative to the remaining patient samples.

The *AC proteome evaluation experiment *resulted in the identification of 199 proteins from pooled AC fluid from 11 patients. In order to identify more AC proteins than the 199 we found in our study, more extensive fractionation of the sample material could have been done, although this is limited by amount of sample material, as well as use of mass spectrometers with higher sequencing capacity and sensitivity. Using lumbar CSF as the basis for comparison with AC fluid is possible when doing a qualitative protein comparison, as proteins are not expected to disappear during migration towards the lumbar area. In a quantitative study between AC fluid and CSF on the other hand, it would be important to use CSF collected in the temporal fossa to avoid effects of the rostro-caudal protein gradients. Of the 199 proteins we identified 15 were not found among the reported 2627 proteins in the CSF database [18] or the 3017 in the plasma database [19]. This does, however, not imply that these proteins are not present in CSF or plasma, as they may not have been previously identified and therefore not added to the database or they could be in the database but under a different accession number, leading to a mismatching. This may explain why we identified 11 of the 15 AC fluid proteins not found in the CSF or plasma databases, in the collected CSF samples, using targeted identification with the MIDAS workflow. Hence the number of proteins that potentially are unique to AC fluid was further decreased to four proteins after this experiment.

The large overlap between AC fluid proteins and CSF (192 of 199 proteins in common) indicates that CSF is important in the filling of the AC. There was much less overlap between identified plasma proteins and AC fluid proteins, with 104 proteins only identified in AC fluid. As the identified protein content of AC almost completely overlapped with the proteins previously identified in CSF, it was not surprising that the biological function annotation of the identified AC fluid also corresponded well with the annotations of the CSF proteins (Figure 8). We did not observe that any particular protein group was not present in AC fluid compared to CSF. In addition small (9.3 kDa), large (479.3 kDa), basic (pI 9.96), and acidic (pI 4.35) proteins were observed in AC fluid, indicating that there were no absolute exclusion of proteins with these different characteristics even though the most extreme basic and acidic proteins were not observed. Certain protein groups could be expected to not be present in AC if there was a selective transport mechanism of fluid across the AC membrane. There could, however still be quantitative differences between certain protein groups in CSF and AC fluid, which would then point towards properties in the filling mechanism. Given the high qualitative similarity we found between AC fluid proteins and CSF proteins, a relevant future study would be to do a quantitative protein comparison of the temporal fossa CSF and AC fluid from the same patient collected at the same time. This would give information about which proteins or protein classes that are differentially expressed between AC fluid and CSF in proximity to the AC, possibly giving further indications of the filling mechanism of AC fluid and the origin of the AC fluid.

## Conclusions

The results of this study indicate that AC fluid is homogenous between patients when evaluated by protein content using a label-free semi-quantitative proteomics approach, a finding supporting results from previous experiments regarding clinical chemistry and mRNA. This points towards a similar filling mechanism of the AC for the examined patients. We found that most proteins identified in AC fluid also could be identified in CSF, while plasma had fewer proteins in common with AC fluid. This indicates that CSF has similar properties to AC fluid. We did not find specific groups of proteins with given properties absent from AC fluid, but there could still be different quantitative trends between CSF and AC fluid. A future quantitative proteomics comparison between CSF and AC collected from the same patients at the same time would reveal this information.

## Abbreviations

AC: arachnoid cyst; ACN: acetonitrile; AM: arachnoid mater; CSF: cerebrospinal fluid; DTT: dithiothreitol; FA: formic acid; HPLC: high pressure liquid chromatography; IAA: iodoacetamide; IDA: information dependent acquisition; LC: liquid chromatography; MIDAS: multiple reaction monitoring initiated detection and sequence analysis; MRM: multiple reaction monitoring; MS: mass spectrometer; ND: not detected; PC: principal component; PCA: principal component analysis; PPM: parts per million; SCX: strong cation exchange; TFA: trichloro formic acid; qTOF: quadropole time-of-flight; XIC: extracted ion chromatographic intensity value.

## Competing interests

The authors declare that they have no competing interests.

## Authors' contributions

MB, ACK, KW, RJU, CAH, FSB conceived and designed the experiments. ACK, TTA, JAO, EO performed the experiments. MB, ACK, ØAH, TTA analyzed the data. KW, CAH operated on the patients. MB performed sample collection and handling. MB, ACK, ØAH, TTA, JAO, EO, KW, RJU, CAH, FSB wrote the paper. All authors read and approved the final manuscript.

## Supplementary Material

Additional file 1 **List of 199 proteins detected in a pool of AC fluid from 11 patients, with number of peptides and sequence coverage**. Proteins are denoted by lead protein IPI accession number. Click here for file

## References

[B1] Morris Z Whiteley WN Longstreth WT Jr Weber F Lee YC Tsushima Y Alphs H Ladd SC Warlow C Wardlaw JM Al-Shahi Salman R Incidental findings on brain magnetic resonance imaging: systematic review and meta-analysis BMJ 2009 339 b3016 10.1136/bmj.b3016 19687093 PMC2728201

[B2] Vernooij MW Ikram MA Tanghe HL Vincent AJ Hofman A Krestin GP Niessen WJ Breteler MM van der Lugt A Incidental findings on brain MRI in the general population N Engl J Med 2007 357 1821 1828 10.1056/NEJMoa070972 17978290

[B3] Helland CA Lund-Johansen M Wester K Location, sidedness, and sex distribution of intracranial arachnoid cysts in a population-based sample J Neurosurg 2010 113 934 939 10.3171/2009.11.JNS081663 20095787

[B4] Rengachary SS Watanabe I Ultrastructure and pathogenesis of intracranial arachnoid cysts J Neuropathol Exp Neurol 1981 40 61 83 7205328

[B5] Rengachary SS Watanabe I Brackett CE Pathogenesis of intracranial arachnoid cysts Surg Neurol 1978 9 139 144 625699

[B6] Galassi E Tognetti F Gaist G Fagioli L Frank F Frank G CT scan and metrizamide CT cisternography in arachnoid cysts of the middle cranial fossa: classification and pathophysiological aspects Surg Neurol 1982 17 363 369 10.1016/0090-3019(82)90315-9 7089853

[B7] Helland CA Aarhus M Knappskog P Olsson LK Lund-Johansen M Amiry-Moghaddam M Wester K Increased NKCC1 expression in arachnoid cysts supports secretory basis for cyst formation Exp Neurol 2010 224 2 424 10.1016/j.expneurol.2010.05.002 20471979

[B8] Go KG Houthoff HJ Blaauw EH Havinga P Hartsuiker J Arachnoid cysts of the sylvian fissure. Evidence of fluid secretion J Neurosurg 1984 60 803 813 10.3171/jns.1984.60.4.0803 6231356

[B9] Berle M Wester KG Ulvik RJ Kroksveen AC Haaland OA Amiry-Moghaddam M Berven FS Helland CA Arachnoid cysts do not contain cerebrospinal fluid: A comparative chemical analysis of arachnoid cyst fluid and cerebrospinal fluid in adults Cerebrospinal Fluid Res 2010 7 8 10.1186/1743-8454-7-8 20537169 PMC2898803

[B10] Aarhus M Helland CA Lund-Johansen M Wester K Knappskog PM Microarray-based gene expression profiling and DNA copy number variation analysis of temporal fossa arachnoid cysts Cerebrospinal Fluid Res 2010 7 6 10.1186/1743-8454-7-6 20187927 PMC2841093

[B11] Zhu W Smith JW Huang CM Mass spectrometry-based label-free quantitative proteomics J Biomed Biotechnol 2010 2010 840518 19911078 10.1155/2010/840518 PMC2775274

[B12] Sandberg DI McComb JG Krieger MD Chemical analysis of fluid obtained from intracranial arachnoid cysts in pediatric patients J Neurosurg 2005 103 427 432 16302614 10.3171/ped.2005.103.5.0427

[B13] Wester K Helland CA How often do chronic extra-cerebral haematomas occur in patients with intracranial arachnoid cysts? J Neurol Neurosurg Psychiatry 2008 79 72 75 10.1136/jnnp.2007.117358 17488784

[B14] Berven FS Kroksveen AC Berle M Rajalahti T Flikka K Arneberg R Myhr KM Vedeler CA Kvalheim OM Ulvik RJ Pre-analytical influence on the low molecular weight cerebrospinal fluid proteome Proteomics Clin Appl 2007 1 699 711 10.1002/prca.200700126 21136725

[B15] Unwin RD Griffiths JR Whetton AD A sensitive mass spectrometric method for hypothesis-driven detection of peptide post-translational modifications: multiple reaction monitoring-initiated detection and sequencing (MIDAS) Nat Protoc 2009 4 870 877 10.1038/nprot.2009.57 19444244

[B16] http://www.uib.no/rg/probe/publications/software/pklfilemerger

[B17] http://www.proteomesoftware.com

[B18] Schutzer SE Liu T Natelson BH Angel TE Schepmoes AA Purvine SO Hixson KK Lipton MS Camp DG Coyle PK Smith RD Bergquist J Establishing the proteome of normal human cerebrospinal fluid PLoS One 2010 5 e10980 10.1371/journal.pone.0010980 20552007 PMC2881861

[B19] Omenn GS States DJ Adamski M Blackwell TW Menon R Hermjakob H Apweiler R Haab BB Simpson RJ Eddes JS Kapp EA Moritz RL Chan DW Rai AJ Admon A Aebersold R Eng J Hancock WS Hefta SA Meyer H Paik YK Yoo JS Ping P Pounds J Adkins J Qian X Wang R Wasinger V Wu CY Zhao X Overview of the HUPO Plasma Proteome Project: results from the pilot phase with 35 collaborating laboratories and multiple analytical groups, generating a core dataset of 3020 proteins and a publicly-available database Proteomics 2005 5 3226 3245 10.1002/pmic.200500358 16104056

[B20] http://geneontology.org

[B21] Johnson RA Wichern DW Applied Multivariate Statistical Analysis 2001 5 Upper Saddle River, N.J: Prentice Hall

[B22] Meinshausen N Bühlmann P Stability Selection J R Statist Soc B (2010) 2010 72 417 473 10.1111/j.1467-9868.2010.00740.x

[B23] Bühlmann P Yu B Analyzing Bagging The Annals of Statistics 2002 30 927 961

[B24] http://au.expasy.org/tools/pi_tool.html

[B25] Wester K Peculiarities of intracranial arachnoid cysts: location, sidedness, and sex distribution in 126 consecutive patients Neurosurgery 1999 45 775 779 10.1097/00006123-199910000-00008 10515470

