# Supplementary material for: Protein profiling reveals inter-individual protein homogeneity of arachnoid cyst fluid and high qualitative similarity to cerebrospinal fluid
Source: Fluids Barriers CNS. 2011 May 20;8:19. doi: 10.1186/2045-8118-8-19 (PMC3120722; doi:10.1186/2045-8118-8-19)
Supplement: Additional file 1 — List of 199 proteins detected in a pool of AC fluid from 11 patients, with number of peptides and sequence coverage. Proteins are denoted by lead protein IPI accession number. [file 2045-8118-8-19-S1.DOC]

| **Lead Protein IPI number** | **Protein name** | **Number of unique peptides** | **Percentage sequence coverage** |
| --- | --- | --- | --- |
| IPI00001611 | Isoform 1 of Insulin-like growth factor II | 2 | 13.90 % |
| IPI00001662 | OPCML protein | 2 | 8.76 % |
| IPI00001952 | Endonuclease domain-containing 1 protein | 4 | 12.00 % |
| IPI00002147 | Chitinase-3-like protein 1 | 2 | 6.01 % |
| IPI00002280 | ProSAAS | 2 | 10.80 % |
| IPI00002714 | cDNA FLJ52545, highly similar to Dickkopf-related protein 3 | 6 | 22.30 % |
| IPI00002925 | Cocaine- and amphetamine-regulated transcript protein | 2 | 18.10 % |
| IPI00003351 | Isoform 1 of Extracellular matrix protein 1 | 4 | 7.78 % |
| IPI00003362 | HSPA5 protein | 2 | 3.66 % |
| IPI00003813 | Isoform 1 of Cell adhesion molecule 1 | 2 | 4.30 % |
| IPI00003919 | Isoform 1 of Glutaminyl-peptide cyclotransferase | 2 | 6.93 % |
| IPI00004440 | Receptor-type tyrosine-protein phosphatase-like N | 2 | 3.06 % |
| IPI00004656 | Beta-2-microglobulin | 2 | 16.80 % |
| IPI00004962 | Golgi integral membrane protein 4 | 2 | 3.59 % |
| IPI00006114 | Pigment epithelium-derived factor | 4 | 12.00 % |
| IPI00006601 | Secretogranin-1 | 10 | 20.80 % |
| IPI00006608 | Isoform APP770 of Amyloid beta A4 protein (Fragment) | 6 | 10.50 % |
| IPI00006662 | Apolipoprotein D | 5 | 28.60 % |
| IPI00007257 | Isoform 2 of Calsyntenin-1 (Fragment) | 6 | 6.18 % |
| IPI00007921 | Isoform 1 of Neurexin-2-alpha | 8 | 5.49 % |
| IPI00008318 | Ephrin type-A receptor 4 | 5 | 7.61 % |
| IPI00009028 | Tetranectin | 6 | 39.10 % |
| IPI00009362 | Secretogranin-2 | 8 | 15.90 % |
| IPI00009950 | Vesicular integral-membrane protein VIP36 | 3 | 8.43 % |
| IPI00009997 | N-acetyllactosaminide beta-1,3-N-acetylglucosaminyltransferase | 4 | 8.43 % |
| IPI00011140 | Protein NOV homolog | 2 | 7.28 % |
| IPI00011218 | Macrophage colony-stimulating factor 1 receptor | 3 | 3.70 % |
| IPI00011229 | Cathepsin D | 5 | 17.50 % |
| IPI00011302 | CD59 glycoprotein | 2 | 18.80 % |
| IPI00011651 | Isoform 1 of Receptor-type tyrosine-protein phosphatase gamma | 2 | 1.59 % |
| IPI00011732 | Isoform 1 of GDNF family receptor alpha-2 | 2 | 5.60 % |
| IPI00012303 | Selenium binding protein 1 | 2 | 4.09 % |
| IPI00012503 | Isoform Sap-mu-0 of Proactivator polypeptide | 5 | 8.78 % |
| IPI00013179 | Prostaglandin-H2 D-isomerase | 6 | 28.90 % |
| IPI00013303 | Limbic system-associated membrane protein | 3 | 10.10 % |
| IPI00014048 | Ribonuclease pancreatic | 2 | 17.90 % |
| IPI00014572 | Secreted protein, acidic, cysteine-rich (Osteonectin), isoform CRA_a | 3 | 10.80 % |
| IPI00015102 | Isoform 1 of CD166 antigen | 4 | 8.92 % |
| IPI00015260 | Protein kinase C-binding protein NELL2 | 10 | 13.40 % |
| IPI00015881 | Isoform 1 of Macrophage colony-stimulating factor 1 | 2 | 4.87 % |
| IPI00016150 | Neuroserpin | 3 | 7.80 % |
| IPI00016334 | Isoform 1 of Cell surface glycoprotein MUC18 | 2 | 4.18 % |
| IPI00016915 | Insulin-like growth factor-binding protein 7 | 4 | 21.30 % |
| IPI00017601 | Ceruloplasmin | 7 | 8.45 % |
| IPI00017696 | Complement C1s subcomponent | 7 | 14.50 % |
| IPI00018136 | Isoform 1 of Vascular cell adhesion protein 1 | 2 | 3.52 % |
| IPI00018276 | Isoform 3 of Seizure 6-like protein 2 | 2 | 2.93 % |
| IPI00019568 | Prothrombin (Fragment) | 5 | 11.40 % |
| IPI00019576 | Coagulation factor X | 2 | 4.71 % |
| IPI00019580 | Plasminogen | 3 | 4.69 % |
| IPI00019591 | cDNA FLJ55673, highly similar to Complement factor B | 8 | 7.50 % |
| IPI00019943 | Afamin | 6 | 12.50 % |
| IPI00020091 | Alpha-1-acid glycoprotein 2 | 2 | 14.40 % |
| IPI00020986 | Lumican | 2 | 5.62 % |
| IPI00020996 | Insulin-like growth factor-binding protein complex acid labile subunit | 2 | 4.46 % |
| IPI00021841 | Apolipoprotein A-I | 11 | 38.60 % |
| IPI00021842 | Apolipoprotein E | 12 | 41.60 % |
| IPI00021854 | Apolipoprotein A-II | 2 | 21.00 % |
| IPI00021855 | Apolipoprotein C-I | 2 | 24.10 % |
| IPI00021891 | Isoform Gamma-B of Fibrinogen gamma chain | 3 | 7.73 % |
| IPI00022371 | Histidine-rich glycoprotein | 4 | 8.57 % |
| IPI00022394 | Complement C1q subcomponent subunit C | 4 | 21.20 % |
| IPI00022395 | Complement component C9 | 4 | 10.70 % |
| IPI00022418 | Isoform 1 of Fibronectin | 30 | 18.90 % |
| IPI00022420 | Retinol-binding protein 4 | 4 | 20.90 % |
| IPI00022426 | Protein AMBP | 5 | 22.20 % |
| IPI00022429 | Alpha-1-acid glycoprotein 1 | 5 | 28.90 % |
| IPI00022431 | cDNA FLJ55606, highly similar to Alpha-2-HS-glycoprotein | 3 | 9.70 % |
| IPI00022432 | Transthyretin | 3 | 25.20 % |
| IPI00022463 | Serotransferrin | 32 | 48.00 % |
| IPI00022488 | Hemopexin | 7 | 18.00 % |
| IPI00022608 | Sortilin-related receptor | 4 | 2.26 % |
| IPI00022733 | 45 kDa protein | 4 | 12.10 % |
| IPI00022892 | Thy-1 membrane glycoprotein | 2 | 14.90 % |
| IPI00022895 | Alpha-1B-glycoprotein | 5 | 10.90 % |
| IPI00022937 | 252 kDa protein | 5 | 2.51 % |
| IPI00023648 | Immunoglobulin superfamily containing leucine-rich repeat protein | 3 | 8.41 % |
| IPI00023673 | Galectin-3-binding protein | 3 | 5.64 % |
| IPI00023814 | Isoform 1 of Neogenin | 3 | 2.94 % |
| IPI00023845 | Isoform 1 of Kallikrein-6 | 4 | 21.70 % |
| IPI00024046 | cDNA FLJ52398, highly similar to Cadherin-13 | 6 | 9.61 % |
| IPI00024966 | Contactin-2 | 7 | 9.33 % |
| IPI00025257 | Semaphorin-7A | 5 | 8.86 % |
| IPI00025465 | cDNA FLJ59205, highly similar to Mimecan | 3 | 7.58 % |
| IPI00026154 | cDNA FLJ59211, highly similar to Glucosidase 2 subunit beta | 3 | 5.98 % |
| IPI00026199 | Glutathione peroxidase 3 | 3 | 15.50 % |
| IPI00026314 | Isoform 1 of Gelsolin | 9 | 17.90 % |
| IPI00027310 | Isoform 1 of Multiple epidermal growth factor-like domains protein 8 | 3 | 1.23 % |
| IPI00027703 | Isoform Long of Alpha-mannosidase 2x | 2 | 1.67 % |
| IPI00027780 | 72 kDa type IV collagenase | 2 | 3.64 % |
| IPI00027827 | Extracellular superoxide dismutase [Cu-Zn] | 4 | 20.00 % |
| IPI00028911 | Dystroglycan | 3 | 4.69 % |
| IPI00029260 | Monocyte differentiation antigen CD14 | 3 | 13.10 % |
| IPI00029658 | Isoform 1 of EGF-containing fibulin-like extracellular matrix protein 1 | 9 | 22.10 % |
| IPI00029739 | Isoform 1 of Complement factor H | 14 | 15.30 % |
| IPI00029751 | Isoform 1 of Contactin-1 | 12 | 14.50 % |
| IPI00031121 | Carboxypeptidase E precursor | 2 | 4.55 % |
| IPI00032179 | Antithrombin-III | 8 | 23.10 % |
| IPI00032220 | Angiotensinogen | 8 | 21.00 % |
| IPI00032291 | Complement C5 | 2 | 1.37 % |
| IPI00032293 | Cystatin-C | 6 | 34.90 % |
| IPI00032328 | Isoform HMW of Kininogen-1 | 3 | 5.28 % |
| IPI00056478 | Isoform 1 of Immunoglobulin superfamily member 8 | 2 | 4.73 % |
| IPI00064667 | Beta-Ala-His dipeptidase | 9 | 18.70 % |
| IPI00102543 | SLIT and NTRK-like protein 1 | 3 | 4.60 % |
| IPI00154742 | IGL@ protein | 4 | 24.80 % |
| IPI00156171 | Isoform 1 of Ectonucleotide pyrophosphatase/phosphodiesterase family member 2 | 4 | 5.91 % |
| IPI00162547 | latrophilin 3 precursor | 2 | 1.57 % |
| IPI00176221 | Neuronal growth regulator 1 | 3 | 13.80 % |
| IPI00177543 | Isoform 5 of Peptidyl-glycine alpha-amidating monooxygenase | 6 | 7.49 % |
| IPI00178854 | Isoform 1 of Contactin-4 | 2 | 2.24 % |
| IPI00183445 | Isoform 1 of Latrophilin-1 | 2 | 1.63 % |
| IPI00216250 | Cell recognition protein CASPR4 | 2 | 1.75 % |
| IPI00217966 | Isoform 1 of L-lactate dehydrogenase A chain | 3 | 10.80 % |
| IPI00218192 | Isoform 2 of Inter-alpha-trypsin inhibitor heavy chain H4 | 6 | 8.64 % |
| IPI00218413 | Biotinidase | 4 | 8.29 % |
| IPI00218733 | Superoxide dismutase [Cu-Zn] | 2 | 16.90 % |
| IPI00218803 | Isoform B of Fibulin-1 | 9 | 17.80 % |
| IPI00219018 | Glyceraldehyde-3-phosphate dehydrogenase | 3 | 13.40 % |
| IPI00219446 | Phosphatidylethanolamine-binding protein 1 | 2 | 18.20 % |
| IPI00219664 | Isoform 2 of Myelin-oligodendrocyte glycoprotein | 2 | 11.30 % |
| IPI00220562 | Neuronal pentraxin-1 | 4 | 9.49 % |
| IPI00220644 | Isoform M1 of Pyruvate kinase isozymes M1/M2 | 5 | 13.90 % |
| IPI00241562 | reelin isoform a | 3 | 1.16 % |
| IPI00289501 | Neurosecretory protein VGF | 5 | 11.90 % |
| IPI00289831 | Isoform PTPS of Receptor-type tyrosine-protein phosphatase S | 2 | 0.98 % |
| IPI00290085 | Cadherin-2 | 4 | 5.41 % |
| IPI00290315 | Chromogranin-A | 7 | 24.50 % |
| IPI00290856 | Lymphatic vessel endothelial hyaluronic acid receptor 1 | 2 | 5.59 % |
| IPI00291136 | Collagen alpha-1(VI) chain | 3 | 3.11 % |
| IPI00291262 | Isoform 1 of Clusterin | 9 | 20.50 % |
| IPI00291866 | Plasma protease C1 inhibitor | 6 | 14.00 % |
| IPI00291867 | Complement factor I | 4 | 9.78 % |
| IPI00292071 | Secretogranin-3 | 5 | 8.55 % |
| IPI00292530 | Inter-alpha-trypsin inhibitor heavy chain H1 | 2 | 2.74 % |
| IPI00292946 | Thyroxine-binding globulin | 3 | 8.43 % |
| IPI00292950 | Serpin peptidase inhibitor, clade D (Heparin cofactor), member 1 | 6 | 9.68 % |
| IPI00296165 | cDNA FLJ54471, highly similar to Complement C1r subcomponent | 6 | 10.60 % |
| IPI00296608 | Complement component C7 | 7 | 10.30 % |
| IPI00296777 | SPARC-like protein 1 | 11 | 28.00 % |
| IPI00297188 | Isoform 3 of Brain-specific angiogenesis inhibitor 2 | 2 | 1.27 % |
| IPI00297284 | insulin-like growth factor-binding protein 2 precursor | 2 | 8.84 % |
| IPI00297646 | Collagen alpha-1(I) chain | 3 | 2.87 % |
| IPI00298388 | Isoform 1 of Phosphoinositide-3-kinase-interacting protein 1 | 2 | 11.40 % |
| IPI00298497 | Fibrinogen beta chain | 5 | 13.80 % |
| IPI00298828 | Beta-2-glycoprotein 1 | 2 | 9.28 % |
| IPI00299024 | Isoform 1 of Brain acid soluble protein 1 | 3 | 20.70 % |
| IPI00299059 | Isoform 2 of Neural cell adhesion molecule L1-like protein | 12 | 14.80 % |
| IPI00299738 | Procollagen C-endopeptidase enhancer 1 | 4 | 12.70 % |
| IPI00300241 | Leucine-rich repeat-containing protein 4B | 2 | 3.37 % |
| IPI00301579 | cDNA FLJ59142, highly similar to Epididymal secretory protein E1 | 2 | 12.90 % |
| IPI00301865 | Isoform 1 of Transmembrane protein 132A | 2 | 2.74 % |
| IPI00302641 | Protocadherin Fat 2 | 3 | 0.92 % |
| IPI00303963 | Complement C2 (Fragment) | 2 | 5.05 % |
| IPI00304273 | Apolipoprotein A-IV | 9 | 24.70 % |
| IPI00305461 | Inter-alpha (Globulin) inhibitor H2, isoform CRA_a | 5 | 6.86 % |
| IPI00306339 | osteopontin isoform b precursor | 5 | 21.30 % |
| IPI00328609 | Kallistatin | 2 | 3.98 % |
| IPI00328746 | Reticulon-4 receptor-like 2 | 3 | 7.86 % |
| IPI00332887 | signal-regulatory protein alpha precursor | 2 | 5.36 % |
| IPI00333776 | Isoform 1 of Neuronal cell adhesion molecule | 14 | 13.70 % |
| IPI00334238 | Neuronal pentraxin receptor | 2 | 8.20 % |
| IPI00334282 | Protein FAM3C | 4 | 26.00 % |
| IPI00337548 | Cell growth regulator with EF hand domain protein 1 | 2 | 12.30 % |
| IPI00374563 | Agrin | 3 | 1.96 % |
| IPI00376427 | Neural cell adhesion molecule 2 | 4 | 5.97 % |
| IPI00384998 | Isoform 7 of Neurofascin | 6 | 5.57 % |
| IPI00386879 | cDNA FLJ14473 fis, clone MAMMA1001080, highly similar to Homo sapiens SNC73 protein (SNC73) mRNA | 5 | 14.60 % |
| IPI00395488 | Vasorin | 2 | 3.71 % |
| IPI00410714 | Hemoglobin subunit alpha | 2 | 16.90 % |
| IPI00414249 | Isoform 1 of Neurexin-3-alpha | 5 | 4.02 % |
| IPI00418163 | complement component 4B preproprotein | 24 | 17.70 % |
| IPI00418262 | Fructose-bisphosphate aldolase | 2 | 5.10 % |
| IPI00426051 | Putative uncharacterized protein DKFZp686C15213 | 2 | 12.50 % |
| IPI00435020 | Isoform 2 of Neural cell adhesion molecule 1 | 9 | 13.20 % |
| IPI00442294 | neurotrimin isoform 3 | 2 | 9.30 % |
| IPI00451624 | Isoform 1 of Cartilage acidic protein 1 | 6 | 11.50 % |
| IPI00456623 | Isoform 1 of Brevican core protein | 5 | 8.67 % |
| IPI00470535 | Dihydropyridine receptor alpha 2 subunit | 5 | 5.95 % |
| IPI00470716 | Isoform 2 of Neuroendocrine protein 7B2 | 4 | 28.00 % |
| IPI00477747 | Isoform 1 of Follistatin-related protein 4 | 2 | 2.02 % |
| IPI00477992 | complement component 1, q subcomponent, B chain precursor | 2 | 11.50 % |
| IPI00478003 | Alpha-2-macroglobulin | 24 | 21.40 % |
| IPI00479708 | Full-length cDNA clone CS0DD006YL02 of Neuroblastoma of Homo sapiens | 2 | 7.47 % |
| IPI00550991 | cDNA FLJ35730 fis, clone TESTI2003131, highly similar to ALPHA-1-ANTICHYMOTRYPSIN | 7 | 19.00 % |
| IPI00553177 | Isoform 1 of Alpha-1-antitrypsin | 10 | 32.30 % |
| IPI00555812 | Isoform 1 of Vitamin D-binding protein | 7 | 21.90 % |
| IPI00607600 | amyloid precursor-like protein 1 isoform 1 precursor | 7 | 12.00 % |
| IPI00641737 | Haptoglobin | 12 | 25.70 % |
| IPI00645363 | Putative uncharacterized protein DKFZp686P15220 | 7 | 17.60 % |
| IPI00646304 | Peptidyl-prolyl cis-trans isomerase B | 2 | 9.72 % |
| IPI00654755 | Hemoglobin subunit beta | 2 | 15.00 % |
| IPI00748312 | Isoform Long of Receptor-type tyrosine-protein phosphatase zeta | 3 | 1.73 % |
| IPI00783287 | Immunglobulin heavy chain variable region (Fragment) | 2 | 32.20 % |
| IPI00783987 | Complement C3 (Fragment) | 36 | 27.10 % |
| IPI00784119 | V-type proton ATPase subunit S1 | 3 | 8.51 % |
| IPI00784985 | IGK@ protein | 6 | 43.00 % |
| IPI00828156 | NANUC-1 heavy chain (Fragment) | 2 | 27.30 % |
| IPI00855821 | neurexin 1 isoform alpha2 precursor | 2 | 3.62 % |
